# Transcatheter Mitral Valve Repair for Failed Surgical Mitral Valve Repair: A Systematic Review and Meta-Analysis

**DOI:** 10.31083/j.rcm2310332

**Published:** 2022-09-28

**Authors:** Hang Xu, Wu Song, Sheng Liu, Zhaoji Zhong

**Affiliations:** ^1^Department of Cardiovascular Surgery, Fuwai Hospital, National Clinical Research Center for Cardiovascular Diseases, National Center for Cardiovascular Diseases, Chinese Academy of Medical Sciences and Peking Union Medical College Chinese Academy of Medical Science, 100037 Beijing, China

**Keywords:** mitral valve repair, failure, recurrence, transcatheter mitral valve repair, MitraClip, Neochord

## Abstract

**Objectives:**

To assess the outcomes of transcatheter mitral valve repair (TMVr) for failed previous surgical mitral valve repair (MVr).

**Methods:**

We searched Pubmed, Embase, and Cochrane Library databases for studies that reported the outcomes of TMVr for failed initial surgical MVr. Data were extracted by 2 independent investigators and subjected to meta-analysis. The 95% confidence interval (CI) was calculated for preoperative demographics, peri-operative outcomes, and follow-up outcomes using binary and continuous data from single-arm studies.

**Results:**

Eight single-arm studies were included, with a total of 212 patients, and mean follow-up ranged from 1.0 to 15.9 months. The pooled rate of residual procedural mitral regurgitation ≤mild was 76% (95% CI: 67%~84%; $I^{2}$ = 0%; 7 studies, 199 patients). During follow-up, mitral regurgitation ≤mild was found in 68% of patients (95% CI: 52%~82%; $I^{2}$ = 57%; 6 studies, 147 patients). Follow-up survival was 94% (95% CI: 88%~98%; $I^{2}$ = 0%; 7 studies, 196 patients). 83% patients (95% CI: 75%~89%; $I^{2}$ = 47%; 6 studies, 148 patients) were in NYHA class I or II.

**Conclusions:**

TMVr for failed surgical MVr was safe and effective, which should be recommended in selected patients if technically feasible.

## 1. Introduction

Mitral valve repair (MVr) is the treatment of choice for severe symptomatic mitral regurgitation (MR) recommended by current guidelines, especially for degenerative mitral valve (MV) disease [1, 2]. However, MVr carries a potential risk for reoperation, reducing late survival [3]. Redo surgery including MVr and mitral valve replacement (MVR) has been the gold standard for failed surgical MVr, defined by the recurrence of moderate or severe MR, mitral valve re-operation for any reason, such as mitral regurgitation, stenosis, hemolysis, or infective endocarditis [4]. However, it is associated with increased technical difficulty inherent to reoperations and greater frailty of the patients [5, 6].

Transcatheter procedures provide a minimally invasive alternative to redo surgery in high-risk patients. For this challenging scenario, transcatheter mitral valve replacement (TMVR) using valve-in-valve (ViV) and valve-in-ring (ViR) techniques have been focused on in the past few years [7, 8, 9, 10]. However, since first reported by Lim *et al*. [11] in 2010, there have been very few studies reporting transcatheter mitral valve repair (TMVr) for failed MVr. The safety and effectiveness of TMVr for failed surgical MVr have not been fully established. Also, there have been no clinical trials comparing TMVr, TMVR, or redo surgery for these patients. Whether the advantages of re-repair compared with Redo MVR can be applied in transcatheter procedures is not clear yet.

Thus, we conducted the present systematic review and meta-analysis to assess the outcomes of TMVr for failed previous surgical MVr.

## 2. Methods

### 2.1 Search Strategy

The study was performed according to the Preferred Reporting Items for Systematic Reviews and Meta-Analyses (PRISMA) guidelines [12]. On May 29, 2022, a comprehensive literature search was conducted of the Pubmed, Embase, and Cochrane Library databases, for relevant studies reporting the outcomes of TMVr for failed surgical MVr. The search strategy is the following words in full text: ((failed) OR (recurrent)) AND ((mitral valve repair) OR (mitral regurgitation) OR (mitral annuloplasty) OR (ring)) AND ((transcatheter) OR (percutaneous) OR (Mitraclip) OR (Neochord)).

The study protocol was registered with PROSPERO, ID: CRD42022336807.

### 2.2 Study Selection

The studies were considered for inclusion if they met the following criteria: (1) the population consisted of patients with previous surgical MVr; (2) re-intervention due to mitral repair failure; (3) previous surgical MVr either with or without an annuloplasty ring; (4) techniques of TMVr were not restricted: transcatheter mitral annuloplasty, edge-to-edge repair, and chordal implantation were acceptable.

The exclusion criteria were: (1) the initial surgery included transcatheter procedures or surgical MVR; (2) re-intervention due to causes other than repair failure; (3) TMVR procedures; (4) open cardiac reoperation; (5) studies with <5 patients included, duplicate publications and review articles.

Three authors (HX, SL, ZZ) screened and assessed the studies independently for inclusion. Disagreements regarding inclusion were resolved via a group consensus.

### 2.3 Data Extraction

Two authors (WS, ZZ) reviewed and extracted the reported data from the studies, which included: details of the study (study design, inclusion criteria, study period, follow-up duration); baseline demographics; procedural details (echocardiographic evaluation of MR and stenosis); perioperative details (major morbidities, mortality, hospital stay); follow-up outcomes (follow-up duration, regurgitation recurrence, mortality, functional status).

### 2.4 Quality Assessment

The study quality and risk of bias were assessed using the methodological index for non-randomized studies (MINORS) [13]. Disagreements were resolved by consensus.

### 2.5 Statistical Analysis and Meta-Analysis

The analyses were performed utilizing R software version 4.2.0 (The R Foundation for Statistical Computing) with the open-source package Meta version 5.2-0, Metamedian version 0.1.5, and Metafor version 3.4-0. Both R and the packages were available as free software released under GNU General Public Licenses. The R software was developed by the R Foundation, downloaded from “https://www.r-project.org/”, the packages were downloaded from the Comprehensive R Archive Network (CRAN) within R.

Statistical heterogeneity was assessed using $I^{2}$. When $I^{2}$≥50%, random-effects models were used. When different percutaneous device used, random effect models were also used. Publication bias was evaluated by constructing funnel plots regarding individual outcomes [14]. For single-arm meta-analysis of binary data, generic inverse variance methods and Freeman-Tukey double arcsine transformation were used. For a single-arm meta-analysis of continuous data, the overall mean and median were calculated utilizing the inverse variance methods. Forest plots were generated to present the pooled results. *p*-value ≤ 0.05 was considered statistically significant.

## 3. Results

### 3.1 Study Selection

**Fig. 1.**
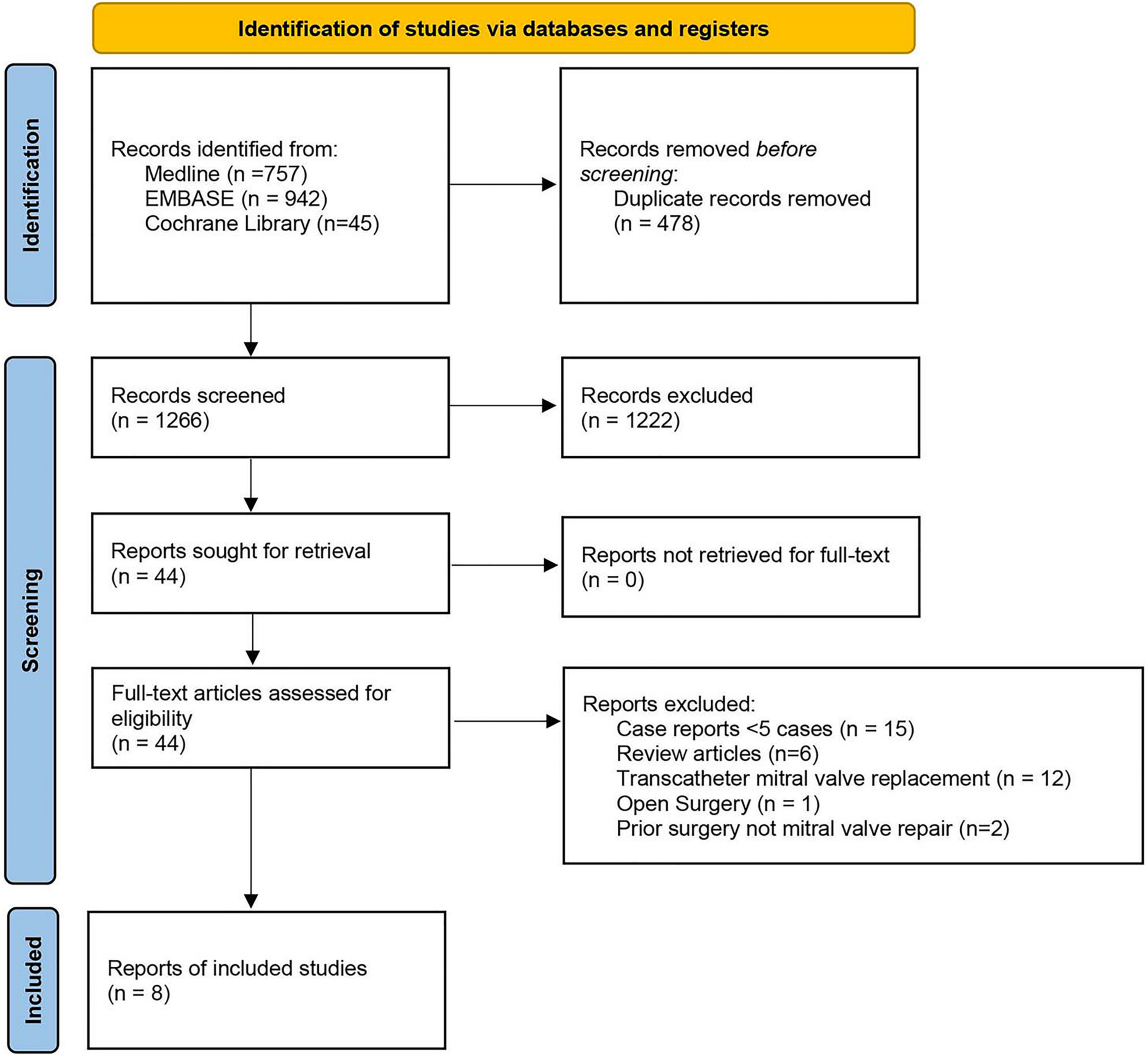
**PRISMA Flow chart**. The selection of studies included in the meta-analysis.

A total of 1266 studies were identified utilizing the search criteria. Based on title and abstract, 44 studies were retrieved for full-text review. TMVR was reported in 12 studies and open cardiac redo surgery in one study. In 2 studies, the previous surgery included MVR or non-mitral cardiac surgery. There were 6 review articles and 15 case reports with less than 5 cases. The remaining 8 studies [15, 16, 17, 18, 19, 20, 21, 22] comprised the pooled data (Fig. 1).

### 3.2 Study Characteristics

Two studies were single-arm prospective studies and 6 were single-arm retrospective studies. Two studies included only degenerative MR, two studies included only functional (Carpentier IIIb) MR and the other 4 did not specify pathological types. A total of 212 patients were included, with 197 patients undergoing MitraClip and 15 undergoing NeoChord. There was no study reporting percutaneous direct annuloplasty for failed surgical MVr. All studies reported short-term follow-up results; mean follow-up ranged from 1 to 15.9 months. The basic characteristics of the studies were listed in Table 1 (Ref. [15, 16, 17, 18, 19, 20, 21, 22]).

**Table 1.** **Basic characteristics of included studies**.

	Study Period	Center	Country	Pathology	Patients	Technique	Mean Follow-up (m)	Conclusion
Rahhab 2021 [22]	2009–2017	Multi-center International	International	Not specified	104	MitraClip	N/A	MitraClip is safe and less invasive
Gerosa 2021 [21]	2014–2018	Multi-center European	European	Degenerative	15	Neochord	1.5 ± 1.2	Selected patients can be treated successfully with Neochord
Niikura 2019 [20]	N/A	Abbott Northwestern Hospital	U.S.	Degenerative	12	MitraClip	18.5 ± 13.1	TMVr with MitraClip is effective, in properly selected patients without mitral stenosis
Pleger 2019 [19]	2013–2018	University Hospital Heidelberg	Germany	Not specified	7	MitraClip	1 ± 0	MitraClip-in-ring is feasible and safe
Braun 2017 [18]	2010–2016	University of Munich	Germany	Not specified	57	MitraClip	15.9 ± 15.5	MitraClip is an alternative for high-risk patients, especially when valve-in-ring is not possible
Saji 2016 [17]	2007–2013	University of Virginia	U. S.	Degenerative and functional	5	MitraClip	7.1 ± 5.2	MitraClip assisted by intracardiac echocardiography is feasible in patients with prior surgical rings
Estévez-Loureiro 2016 [16]	2010–2015	Complejo Asistencial Universitario de León	Spain	Degenerative and functional	6	MitraClip	11.1 ± 10.8	MitraClip is safe and effective following surgical annuloplasty
Grasso 2014 [15]	2008–2013	Ferrarotto Hospital	Italy	Funcional	6	MitraClip	12.8 ± 10.9	MitraClip is safe and effective in patients with an annuloplasty ring

### 3.3 Methodological Quality Assessment

The studies scored 7–11 out of 24 on the MINORS index, losing points mainly for lack of prospective data collection, unbiased endpoint assessment, and study size calculation. Quality assessment of included studies was listed in Table 2 (Ref. [15, 16, 17, 18, 19, 20, 21, 22]). Funnel plot analysis (**Supplementary Figs. 1,2**) did not suggest potential publication bias.

**Table 2.** **Quality assessment of included studies using the ^†^MINORS index**.

	Rahhab 2021 [22]	Gerosa 2021 [21]	Niikura 2019 [20]	Pleger 2019 [19]	Braun 2017 [18]	Estévez-Loureiro 2016 [17]	Saji 2016 [16]	Grasso 2014 [15]
A clearly stated aim	2	2	1	2	2	1	1	1
Inclusion of consecutive patients	2	2	0	0	0	0	2	2
Prospective collection of data	0	2	0	0	0	0	0	2
Endpoints appropriate to the aim of the study	2	2	2	2	2	2	2	2
Unbiased assessment of the study endpoint	0	0	0	0	0	0	0	0
Follow-up period appropriate to the aim of the study	2	1	2	2	2	2	2	2
Loss to follow up less than 5%	2	2	2	2	2	2	2	2
Prospective calculation of the study size	0	0	0	0	0	0	0	0
Additional criteria in the case of comparative study								
An adequate control group								
Contemporary groups								
Baseline equivalence of groups								
Adequate statistical analyses								
Total	10	11	7	8	8	7	9	11

†The items are scored 0 (not reported), 1 (reported but inadequate), or 2 (reported and adequate). The global ideal score being 16 for non-comparative studies and 24 for comparative studies. MINORS: methodological index for non-randomized studies.

### 3.4 Preoperative Demographics

The pooled mean age was 73.5 (95% CI: 70.15%~76.77%; $I^{2}$ = 63%; 8 studies, 212 patients), and 70% (95% CI: 62%~78%; $I^{2}$ = 4%; 7 studies, 155 patients) was male. Median interval between previous surgical MVr and TMVr was 5.0 years (95% CI: 3.7%~6.3%; $I^{2}$ = 51%; 7 studies, 155 patients).

Mean preoperative left ventricular ejection fraction (LVEF) was 42.6% (95% CI: 33.6%~52.0%; $I^{2}$ = 98%; 5 studies, 137 patients). 87% of enrolled patients (95% CI: 75%~97%; $I^{2}$ = 56%; 8 studies, 212 patients) were in New York Heart Association (NYHA) class III and IV.

The operative risk was estimated using the Society of Thoracic Surgeons (STS) score in 6 studies and the EuroSCORE in 3 studies. The pooled STS score was 6.7% (95% CI: 5.1%~8.4%; $I^{2}$ = 88%; 6 studies, 191 patients). The pooled EuroSCORE score was 13.2% (95% CI: 2.7%~23.1%; $I^{2}$ = 95%; 3 studies, 27 patients).

### 3.5 Perioperative Outcomes

**Fig. 2.**
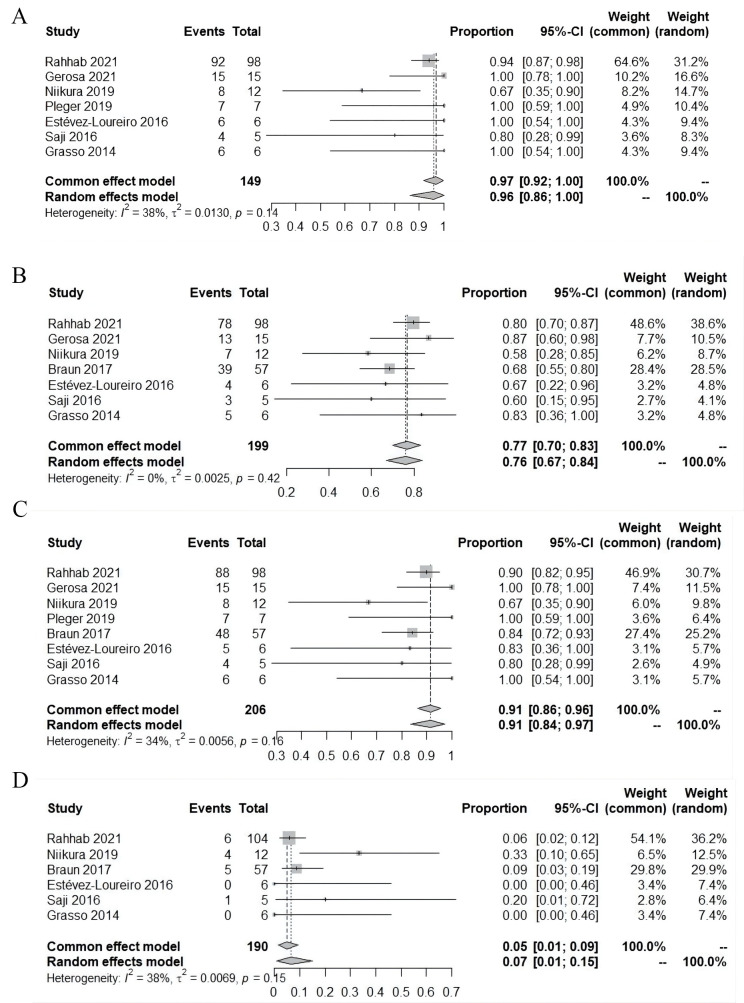
**Perioperative outcomes**. (A) Procedural mitral regurgitation reduction ≥1 grade. (B) Residual procedural mitral regurgitation ≤mild. (C) Residual procedural mitral regurgitation ≤moderate. (D) Significant procedural mitral stenosis. CI, confidence interval.

The pooled rate of procedural MR reduction ≥1 grade was 96% (95% CI: 86%~100%; $I^{2}$ = 38%; 7 studies, 149 patients) (Fig. 2). The pooled rate of residual procedural MR ≤mild was 76% (95% CI: 67%~84%; $I^{2}$ = 0%; 7 studies, 199 patients). The pooled rate of residual procedural MR ≤moderate was 91% (95% CI: 84%~97%; $I^{2}$ = 34%; 8 studies, 206 patients). Significant procedural mitral stenosis was found in 5% patients (95% CI: 1%~9%; $I^{2}$ = 38%; 6 studies, 190 patients).

Perioperative mortality was reported in all studies, including 6 studies with no hospital death. The pooled mortality rate was 0% (95% CI: 0%~1%; $I^{2}$ = 0%; 8 studies, 212 patients). Major perioperative morbidity was reported in only one study, and the morbidity rate was 4.8% (5/104).

Median hospital stay was 4.1 days (95% CI: 2.9%~6.1%; $I^{2}$ = 82%; 6 studies, 145 patients).

### 3.6 Follow-Up Outcomes

Follow-up MR was reported in 6 studies. MR ≤mild was found in 68% patients (95% CI: 52%~82%; $I^{2}$ = 57%; 6 studies, 147 patients), and MR ≤moderate in 90% patients (95% CI: 78%~98%; $I^{2}$ = 58%; 6 studies, 147 patients).

**Fig. 3.**
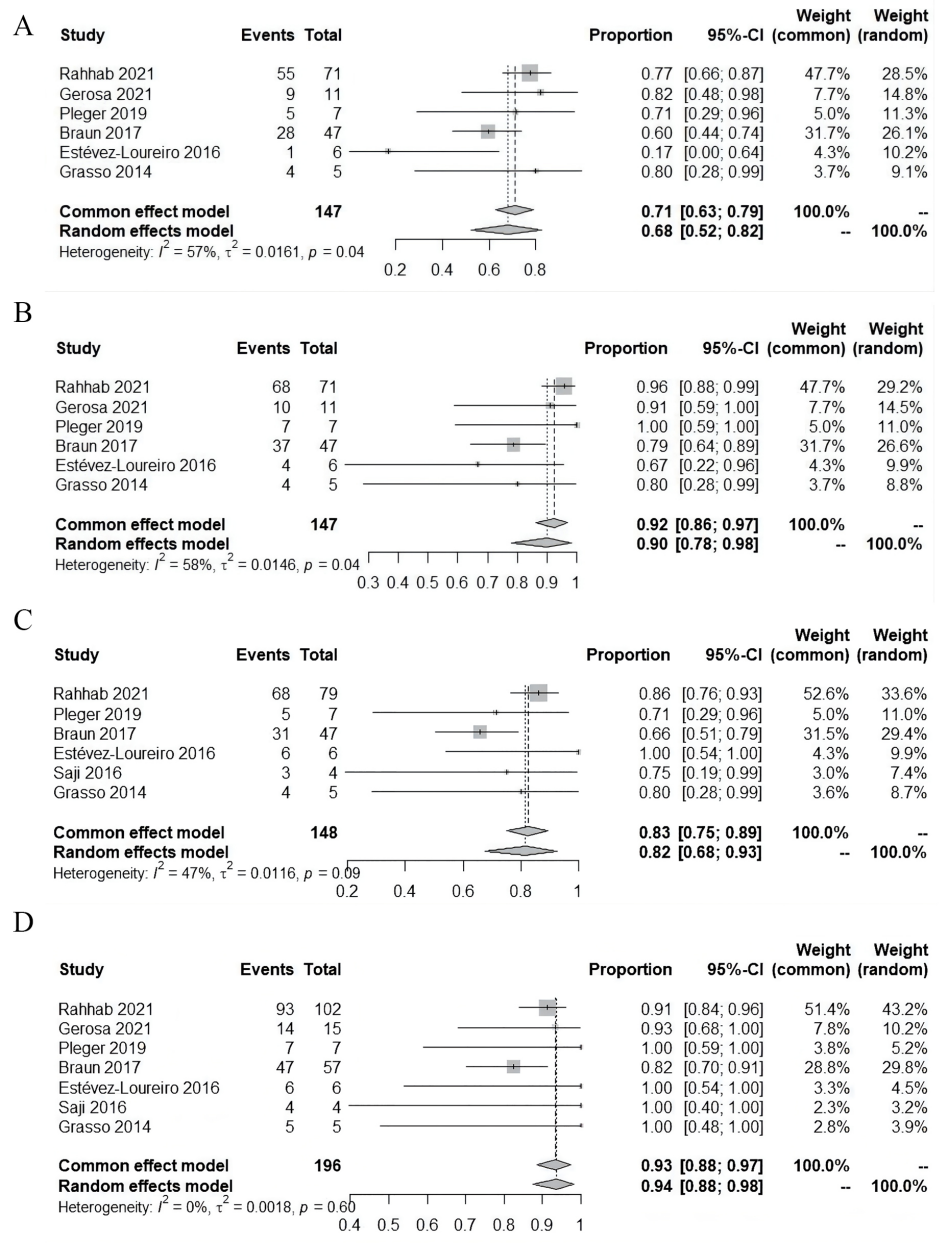
**Follow-up outcomes**. (A) Residual mitral regurgitation ≤mild. (B) Residual mitral regurgitation ≤moderate. (C) NYHA class ≤II. (D) Survival. CI, confidence interval; NYHA, New York Heart Association.

Follow-up survival was reported in 7 studies, and pooled survival was 94% (95% CI: 88%~98%; $I^{2}$ = 0%; 7 studies, 196 patients). In addition, 83% of patients (95% CI: 75%~89%; $I^{2}$ = 47%; 6 studies, 148 patients) were in NYHA class ≤II during follow-up (Fig. 3).

## 4. Discussion

In the present systematic review and meta-analysis, the major finding was that TMVr was safe and effective for failed surgical MVr. For patients who were not a candidate or at high risk for reoperation, TMVr reduced MR and improved functional status less invasively. This was the first systematic review and meta-analysis that focused on the transcatheter repair of failed previous surgical MVr.

### 4.1 TMVr vs. Open Redo Surgery

Redo mitral valve surgery has been the golden standard for failed MVr before the era of percutaneous interventions [4]. However, it was associated with higher perioperative risk. Kwedar *et al*. [5] analyzed early mitral reoperation data from Medicare. The hospital mortality was 9.8% for re-repair, 12.7% for MVR with bioprosthesis, and 12.2% for mechanical prosthesis [5]. Ejiofor *et al*. [6] reported a group of mitral reoperative patients who were eligible for TMVR (ViR or ViV), and the operative mortality was 5% for previous MVr versus 9% for the previous MVR. In retrospective studies focusing on reoperation for failed MVr, the hospital mortality was even lower, especially for re-repair groups. In our study, the pooled hospital mortality for TMVr was less than 1%, lower than predicted by the STS score (6.7%) and EuroSCORE (13.2%). It was relatively low for the group of high-risk patients, some of which with prohibitive medical conditions for redo surgery.

While “optimal” correction of MR was achieved by open cardiac surgery, TMVr sometimes provided only “acceptable” results [23]. Pooled data in our meta-analysis suggested that 96% of patients had ≥1+ reduction of regurgitation and 91% of patients had ≤moderate residual regurgitation. However, when open cardiac surgery was performed, the aim that the surgeon bear in mind was always ≤mild residual regurgitation [24]. If the strict “surgical” standard was applied to TMVr, the pooled success rate was only 76%. Although the effect of mild residual regurgitation on long-term survival was still controversial, moderate residual regurgitation was associated with adverse effects [3, 25]. From this point of view, the effect of TMVr was inferior to surgical re-repair and represents a compromise with minimal invasion. As a novel technique to repair MR, the MitraClip G4 system had been demonstrated to be effective and efficient, especially in cases of moderate to severe MR. However, none of these studies have used this new device for treatment.

The long-term outcome of TMVr for failed MVr has yet to be studied. In the present study, 83% of patients were in NYHA class I or II at follow-up, 90% of patients had ≤moderate regurgitation, and 68% had ≤mild residual regurgitation. The immediate and sustained results of surgical re-repair had been proven to be excellent. At 5 years after surgical re-repair, MR recurrence occurred in less than 10% of patients; freedom from reoperation was 83%~96% and survival was 76%~100% [4]. Considering the short follow-up duration of the pooled studies, the durability of TMVr for failed MVr should be further examined and compared with surgical re-repair. The endpoint of rehospitalization for heart failure was limited in studies included in this meta-analysis, necessitating future studies to examine if TMV affects this endpoint in patients with failed surgical MVr.

TMVr for failed MVr can only be performed in a subset of patients. For example, it was contraindicated in the setting of endocarditis and seldomly used in patients with mitral stenosis. For patients with a small rigid annuloplasty ring or stiff leaflets, elevated transmitral pressure gradient might be a concern [26]. In most cases, the re-repair rate for open surgery was less than half, but Anyanwu *et al*. [27] reported a re-repair rate of 85% (90% for degenerative disease) in an experienced heart center. The feasibility of TMVr is based on an individualized analysis of the MV pathology.

Thus, redo surgery provides definite immediate and long-term results with acceptable perioperative risk for most patients. Re-repair should be preferred to TMVr for appropriately selected patients. For high-risk patients, TMVr is an alternative which reduces MR and improves functional status less invasively.

### 4.2 TMVr vs. ViR-TMVR

For primary MR, the result MVr was superior to MVR, even for complex repair, for elderly patients, and other causes such as papillary muscle rupture and infective endocarditis [1]. For secondary MR, the proof of surgical correction was limited, but different techniques of subvalvular repair were being evaluated. For failed MVr, re-repair was also associated with significantly lower peri-operative mortality and improved late survival compared with MVR [4].

ViR procedures for patients with a prior annuloplasty ring/band have been introduced since the invention of TMVR. Mortality with ViR-TMVR at 30 days is 0%~18%, and 0%~34% at 1 year [8]. Recently, the midterm results of the VIVID registry were evaluated, including 222 ViR patients. Residual stenosis (26.9% severe patient-prosthesis mismatch), residual regurgitation (16.6% ≥moderate), and left ventricular outflow tract (LVOT) obstruction (5.9%) are common after ViR procedures. Residual regurgitation and LVOT obstruction are especially common in ViR compared with ViV procedures (*p *< 0.001). For ViR procedures, 30-day mortality was 8.6%, and the suboptimal survival extended to 4 years with about 50% mortality [10]. From pooled data of our study, TMVr had a lower risk of mitral stenosis (5%) and hospital mortality (0%). Considering the suboptimal results of ViR-TMVR, our study might suggest TMVr was preferable to ViR-TMVR, although no trials were comparing them directly.

The outcomes of the two procedures were influenced by anatomic and technical factors. For example, the discontinuous portions of prior incomplete rings might result in a paravalvular leak. Large septal bulge and small aorto-mitral angle increased the risk of LVOT obstruction [8, 9]. Some of these factors were the intrinsic characteristics of the ViR procedures and were difficult to avoid. For patients with these unfavorable factors, TMVr should be considered as an alternative therapy to ViR-TMVR. The feasibility of TMVr should be analyzed individually, aiming to optimize hemodynamics and device durability.

Thus, TMVr was associated with better immediate and sustained outcomes and should be attempted if technically feasible, especially for those with unfavorable anatomical factors for ViR-TMVR.

### 4.3 Transcatheter Edge-To-Edge Repair vs. Chordal Implantation

Transcatheter edge-to-edge repair has been the most frequently used TMVr procedure. Besides edge-to-edge repair, there was also growing evidence for transcatheter procedures targeting the annulus and the chordae [28]. In open MV re-repair surgery, the frequently used techniques included leaflet resection, ring removal/annuloplasty, edge-to-edge repair, and neochordae [4, 27].

For the choice of TMVr techniques for failed surgical MVr, current literature was limited by a small number of patients and short follow-up. No comparison could be made in this challenging scenario. The use of Neochord procedures might offer several advantages. It was not limited by the presence of an annuloplasty ring and minimizes the risk of mitral stenosis [29]. Compared with MitraClip, it offered more physiological hemodynamics [26] and an annular shape [30]. However, whether these advantages could be translated into improved clinical prognosis has not been determined. For a degenerative disease with the precise site of recurrent prolapse, chordal implantation might be more effective, while for leaflet tethering or annular dilation, edge-to-edge repair might provide a simple but effective choice.

To our knowledge, there has been no study reporting percutaneous mitral annuloplasty for failed surgical MVr, although it has been used for failed MitraClip [31]. Combined transcatheter procedures of concomitant annuloplasty and chordal implantation, have already been performed to treat complex MR [32, 33], but not failed surgical MVr.

In conclusion, there has been a paucity of data on the long-term outcomes comparing transcatheter edge-to-edge and chordal repair. NeoChord might be an alternative in anatomically suitable cases.

### 4.4 Limitations

There were several limitations of our meta-analysis. Firstly, the studies included were all single-arm studies with small sample sizes. Of the studies included, only 2 had decent numbers, and these two appear to sway the results. Meanwhile, no comparison was performed between TMVr and surgical MVr in failed surgical MVr due to lack of research addressing the issue. Secondly, in spite of increased interest, there are limited studies on TMVr after failed surgical MVr. Accordingly, most recent studies are observational and retrospective, with small sample size, a variety of follow-up periods, and different ethnicities of participants. Due to this, studies were scored poorly on the MINORS index, heterogeneous, and biased. Thirdly, there was no uniform standard for selecting redo surgery vs. transcatheter, and TMVr vs. TMVR. The choice was made based on the surgeons or institutional experience. Fourthly, most studies included focused on procedural and in-hospital outcomes, with relatively short follow-up.

## 5. Conclusions

TMVr for failed surgical MVr had encouraging short-term outcomes and should be recommended in selected high-risk and anatomically suitable patients if technically feasible.

## Supplementary Material

Supplementary material associated with this article can be found, in the online version, at https://doi.org/10.31083/j.rcm2310332.
