# Supplementary material for: Transcatheter Mitral Valve Repair for Failed Surgical Mitral Valve Repair: A Systematic Review and Meta-Analysis
Source: Rev Cardiovasc Med. 2022 Sep 28;23(10):332. doi: 10.31083/j.rcm2310332 (PMC11267326; doi:10.31083/j.rcm2310332)
Supplement: Supplementary file 1 [file 2153-8174-23-10-332-s1.zip › 2153-8174-23-10-332-s1/Supplementary figure 1.docx]

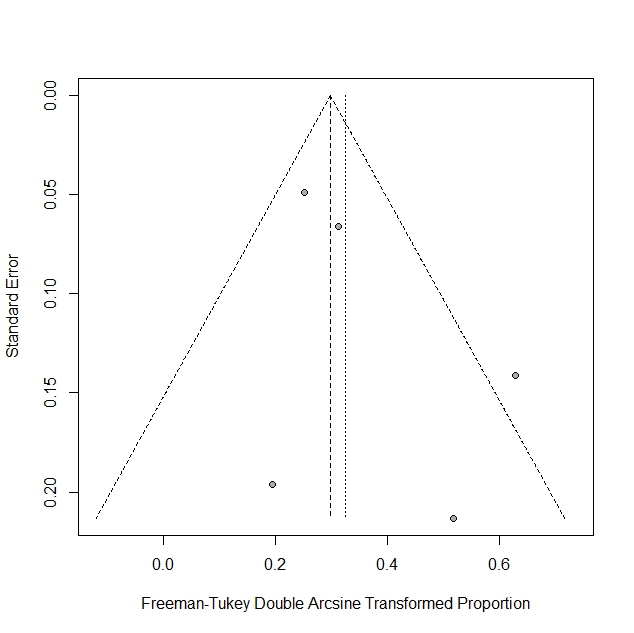

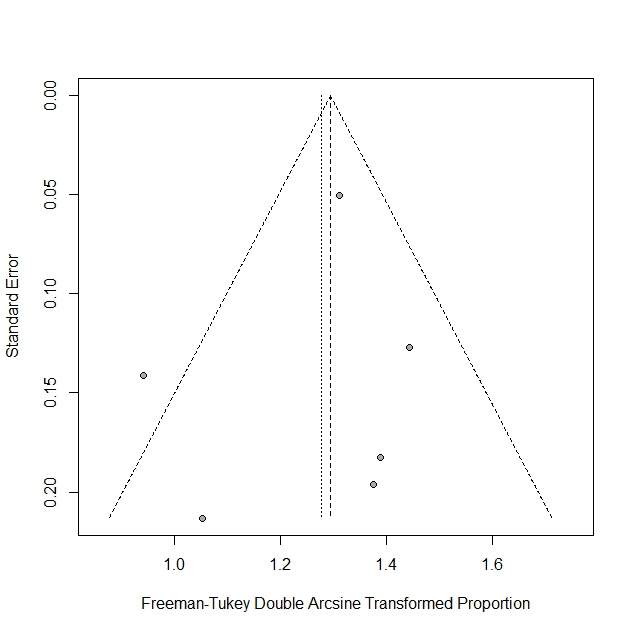

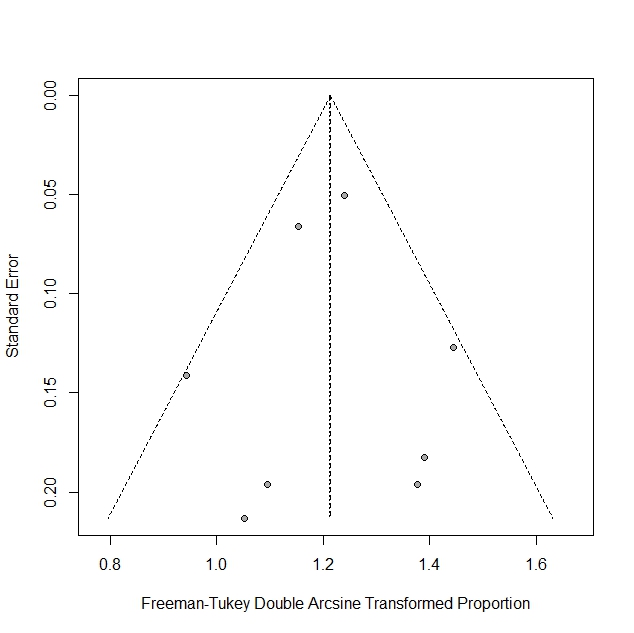

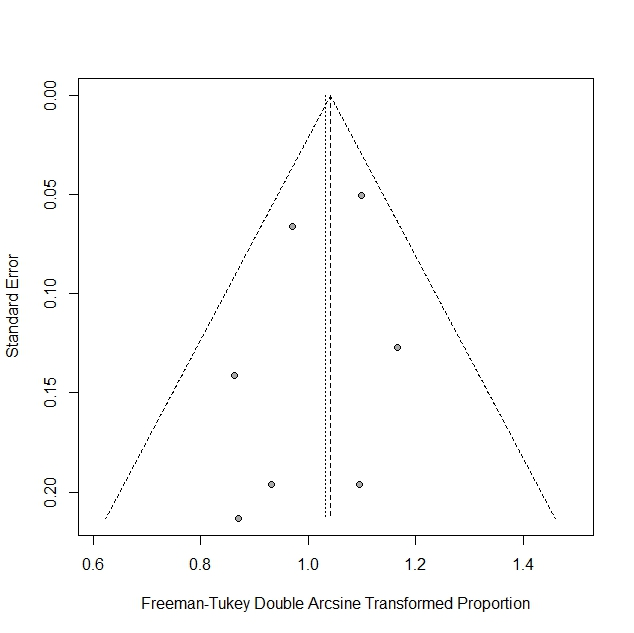


**Supplementary Figure 1 Funnel plot analysis for perioperative outcomes.** (A) Procedural mitral regurgitation reduction ≥ 1 grade; (B) Residual procedural mitral regurgitation ≤ mild; (C) Residual procedural mitral regurgitation ≤ moderate; (D) Significant procedural mitral stenosis.

A

C

B

D
