# Supplementary material for: Transcatheter Mitral Valve Repair for Failed Surgical Mitral Valve Repair: A Systematic Review and Meta-Analysis
Source: Rev Cardiovasc Med. 2022 Sep 28;23(10):332. doi: 10.31083/j.rcm2310332 (PMC11267326; doi:10.31083/j.rcm2310332)
Supplement: Supplementary file 1 [file 2153-8174-23-10-332-s1.zip › 2153-8174-23-10-332-s1/Supplementary figure 2.docx]

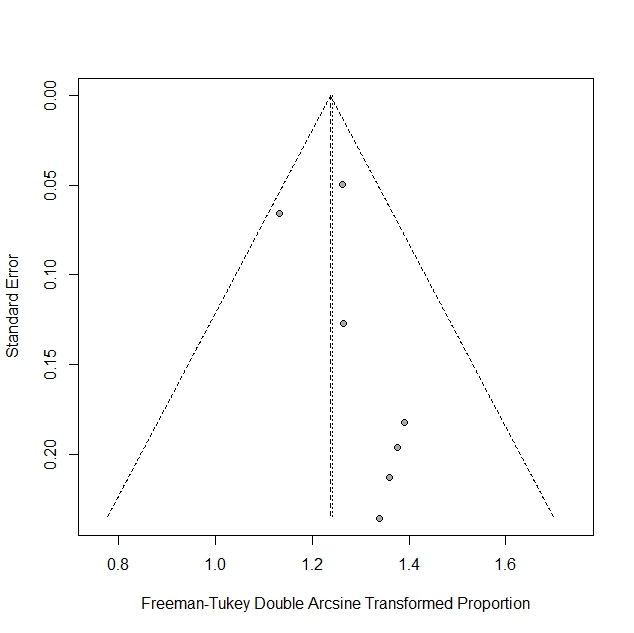

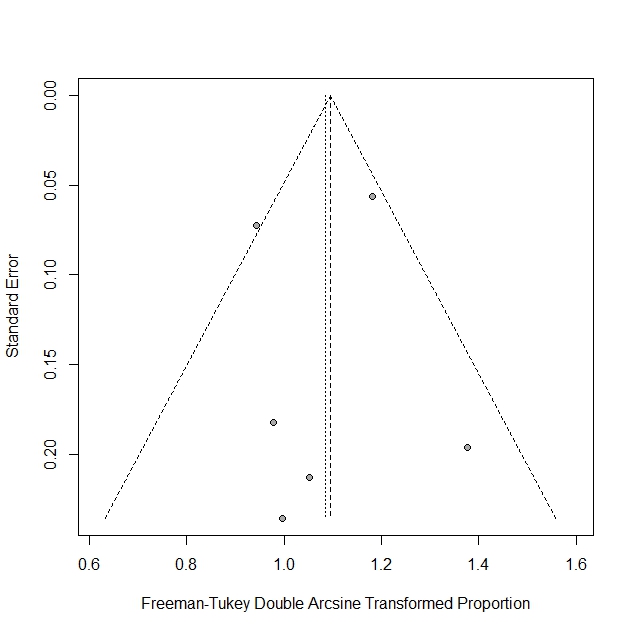

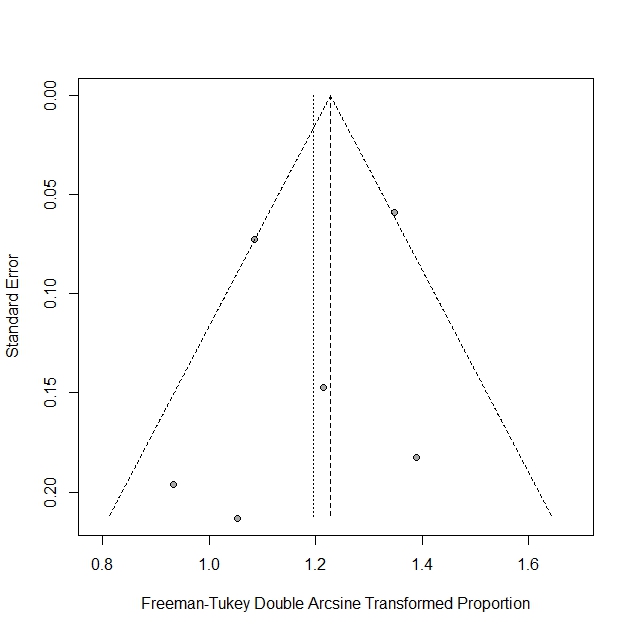

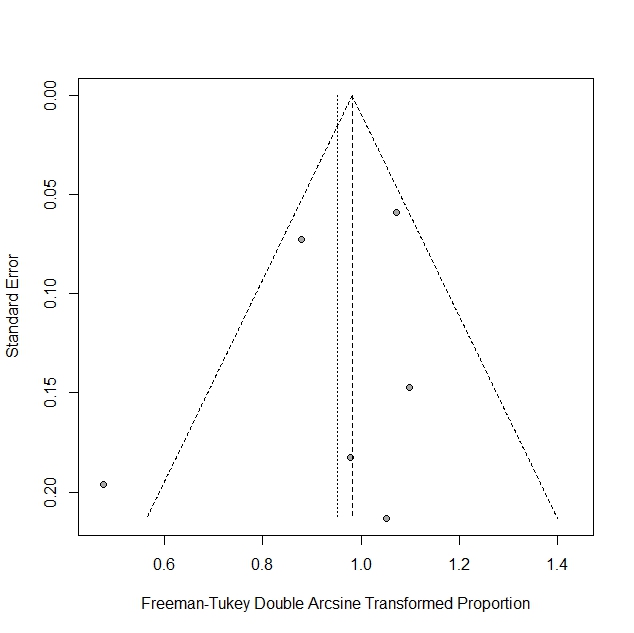


D

C

B

A

**Supplementary Figure 2** **Funnel plot analysis for follow-up outcomes.** (A) Residual mitral regurgitation ≤ mild; (B) Residual mitral regurgitation ≤ moderate; (C) NYHA class ≤ II; (D) Survival. NYHA, New York Heart Association
